# Supplementary material for: Loneliness, Wellbeing, and Social Activity in Scottish Older Adults Resulting from Social Distancing during the COVID-19 Pandemic
Source: Int J Environ Res Public Health. 2021 Apr 24;18(9):4517. doi: 10.3390/ijerph18094517 (PMC8123175; doi:10.3390/ijerph18094517)
Supplement: Supplementary file 1 [file ijerph-18-04517-s001.zip › FileS1.pdf]

Supplementary File 3: Additional qualitative information:

Twenty-seven participants provided very detailed responses of their social activity. The nature of their responses is likely a result of previous questions where participants were asked to report on time spent in social activity, but nonetheless provides a more detailed idea of the variation in social activity and the lived experience:

MONDAY Golf - 120 minutes TUESDAY Golf - 120 minutes THURSDAY Video chat - 30 minutes (not every week) FRIDAY Golf - 120 minutes SATURDAY Video chat - 60 minutes (not every week) SUNDAY Shopping - 90 minutes (most weeks) Golf - 210 minutes (not every week) EVERY DAY Talking with wife - 120 minutes? Being in same room as wife - 480 minutes Total social contact = > 4000 minutes
- Male, 68 years, 'accessible rural'

Monday telephone calls 20 mins Wednesday - telephone calls - 20 mins Thursday walk with friend - 180 mins Friday visit from friend - 90 mins Telephone calls with family and friends-- 180 mins
- Female, 74 years, 'rest of Scotland'

Generally, I hate talking on the phone, would never call for 'a chat' and prefer to write or be physically with my friends or family. Now, I'm a little more comfortable speaking on the phone, especially once I realised some single friends were finding lockdown difficult. I have more phone communication & Facetime with my children & grandchildren. I see Q46 asks about social contact and the examples imply quite long engagements. Most of mine are brief and written - texting or WhatsApp. Little & often, checking in with each other.
- Female, 69 years, 'rest of Scotland'

Tues Zoom coffee morning with work mates 60mins Zoom choir rehearsal 70mins Wed zoom chat with art group 40mins Zoom Pre-dinner drinks with friends 40 mins Thurs Zoom run cancer support group 75 mins Friday Zoom chat with a group I sing with 40 mins Sunday monthly Zoom Facilitate Grief Circle 75mins
- Female, 69 years, 'remote rural'

FaceTime son and grandchildren twice a week total 2 hours. FaceTime daughter and family once a week 1 hour. FaceTime grandson once a week 30 mins plus he phones and gets other shopping. Phone other daughter 1 hour a week. Zoom quiz with family every Saturday night for 1.5 hours. Regular phone calls to/from family. Phone calls weekly to/from sister 1 hour. Phone to/from 2 friends weekly for 1 hour each. Chat in garden to neighbours. Daughter and son in law does shopping have chat when they drops it off.
- Female, 65 years, no postcode provided

Saturday - Family breakfast call 90 minutes Sunday - Church online 60 minutes Chatting after exercise classes 60 minutes (4 days) Spending time with dependant parent 90 minutes (4 days) Daily phone calls with daughter 20 minutes Daily phone calls with partner 30minutes Total social contact time 1100 minutes
- Female, 60 years, 'rest of Scotland'

Twice Daily: FaceTime calls with partner in USA an hour each time Twice a week face to face meetings with friends in garden Twice a week FaceTime calls with other friends further afield Four times a week, phone chat with other friends while I'm walking Occasional zoom parties to celebrate friends' birthdays
- Male, 61 years, 'rest of Scotland'

Getting sent photos and videos of our grandkids by sons. One was born in lockdown only twenty miles away but I only got our first hug when she was 13wks old. That was hard! Another two had birthdays and I only saw photos. Elderly neighbours want to chat but won't socially distance - that's awkward! Lots of friends don't have Internet so text them but lots are depressed with the situation and are withdrawing from communication with anyone. Husband is deaf and he feels isolated. Listen to sons on the phone but they are having terrible employment

implications from the situation, one lost his whole business, so it's impossible to say anything about feeling like you need cheering up yourself! Miss all my grandkids so try to WhatsApp them and reassure them that we are doing well and encouraging them to be positive! Call 90yr old aunt who lives alone and is shielding. She hasn't seen anyone in months and lives ten floors up. So really, there's a lot of people worse off than us! She had her 90th birthday alone, so it was important to call and speak with her. Only a few minutes each day on a few days a week keeping in touch with people.
- Female, 61 years, 'rest of Scotland'
