# Supplementary material for: Loneliness, Wellbeing, and Social Activity in Scottish Older Adults Resulting from Social Distancing during the COVID-19 Pandemic
Source: Int J Environ Res Public Health. 2021 Apr 24;18(9):4517. doi: 10.3390/ijerph18094517 (PMC8123175; doi:10.3390/ijerph18094517)
Supplement: Supplementary file 1 [file ijerph-18-04517-s001.zip › TableS1.pdf]

S2: Supplementary material for Figures 2, 3 and 4 depicting associations with loneliness (UCLA) and wellbeing (EQ5D):

Model convergence

```
> # check model convergence
> conv.check(mod)

Largest absolute gradient value: 1.719205e-06
Observed information matrix is positive definite
Eigenvalue range: [0.2336655,202420832681]

Trust region iterations before smoothing parameter estimation: 7
Loops for smoothing parameter estimation: 50
Trust region iterations within smoothing loops: 151
Estimated overall probability range: 0.001199307 0.9999966
Estimated overall density range: 1.013634e-05 7.587381
```

Model output – overall

```
> # check model summary
> summary(mod)

COPULA:   Clayton
MARGIN 1: gamma
MARGIN 2: gamma

EQUATION 1
Link function for mu.1: log
Formula: UCLAtotal ~ sexbinary + s(age) + s(SIMDquintile, k = 3) + SIMDurbrural3fold +
    factor(PAcat) + ideal_sleeper + s(BPSSQtotal) + s(socialnetworksize) +
    s(socialhourperweek) + s(healthrating) + s(weeklyWALKmin) +
    s(screenhrstruncated)

Parametric coefficients:
                    Estimate Std. Error z value Pr(>|z|)
(Intercept)         2.446927   0.036336  67.341  < 2e-16 ***
sexbinary2          0.077445   0.023292   3.325 0.000884 ***
SIMDurbrural3fold2 -0.041863   0.024753  -1.691 0.090801 .
SIMDurbrural3fold3 -0.009605   0.039099  -0.246 0.805948
factor(PAcat)2     -0.003665   0.034647  -0.106 0.915764
factor(PAcat)3      0.013883   0.036491   0.380 0.703605
ideal_sleeperless   0.024631   0.020395   1.208 0.227161
ideal_sleepermore  -0.028548   0.050059  -0.570 0.568479
---
Signif. codes:  0 ‘***’ 0.001 ‘**’ 0.01 ‘*’ 0.05 ‘.’ 0.1 ‘ ’ 1

Smooth components' approximate significance:
                        edf Ref.df  Chi.sq  p-value
s(age)                3.474  4.326   5.533  0.31412
s(SIMDquintile)       1.000  1.000   0.073  0.78758
s(BPSSQtotal)         3.289  4.084 134.057  < 2e-16 ***
s(socialnetworksize)  8.136  8.708  25.237  0.00157 **
s(socialhourperweek)  1.497  1.833   0.574  0.70896
s(healthrating)       1.478  1.821  30.493 6.49e-08 ***
s(weeklyWALKmin)      6.779  7.835  16.158  0.03866 *
s(screenhrstruncated) 1.000  1.000   1.743  0.18681
---
Signif. codes:  0 ‘***’ 0.001 ‘**’ 0.01 ‘*’ 0.05 ‘.’ 0.1 ‘ ’ 1
EQUATION 2
Link function for mu.2: log
Formula: EQ5Dtotal ~ sexbinary + s(age) + s(SIMDquintile, k = 3) + SIMDurbrural3fold +
    factor(PAcat) + ideal_sleeper + s(BPSSQtotal) + s(socialnetworksize) +
    s(socialhourperweek) + s(healthrating) + s(weeklyWALKmin) +
    s(screenhrstruncated)

Parametric coefficients:
                    Estimate Std. Error z value Pr(>|z|)
(Intercept)         1.885890   0.019135  98.555   <2e-16 ***
sexbinary2          0.027021   0.012413   2.177   0.0295 *
SIMDurbrural3fold2 -0.009627   0.013509  -0.713   0.4761
SIMDurbrural3fold3 -0.040985   0.021543  -1.902   0.0571 .
factor(PAcat)2     -0.035662   0.018109  -1.969   0.0489 *
factor(PAcat)3     -0.043354   0.019314  -2.245   0.0248 *
ideal_sleeperless   0.026309   0.011102   2.370   0.0178 *
ideal_sleepermore   0.003611   0.027599   0.131   0.8959
---
Signif. codes:  0 ‘***’ 0.001 ‘**’ 0.01 ‘*’ 0.05 ‘.’ 0.1 ‘ ’ 1

Smooth components' approximate significance:
                        edf Ref.df  Chi.sq p-value
s(age)                2.322  2.952   6.233 0.07882 .
s(SIMDquintile)       1.000  1.000   7.315 0.00684 **
s(BPSSQtotal)         3.620  4.477  11.890 0.03140 *
s(socialnetworksize)  1.000  1.000   3.477 0.06223 .
s(socialhourperweek)  1.000  1.000   0.018 0.89240
s(healthrating)       5.298  6.396 310.597 < 2e-16 ***
s(weeklyWALKmin)      3.058  3.793  14.430 0.00521 **
s(screenhrstruncated) 1.000  1.000   3.490 0.06173 .
---
Signif. codes:  0 ‘***’ 0.001 ‘**’ 0.01 ‘*’ 0.05 ‘.’ 0.1 ‘ ’ 1

sigma.1 = 0.299(0.287,0.31)  sigma.2 = 0.164(0.159,0.172)
theta = 0.351(0.25,0.483)  tau = 0.149(0.111,0.194)
n = 976  total edf = 63.9
```
