# Supplementary figures and images for: Loneliness, Wellbeing, and Social Activity in Scottish Older Adults Resulting from Social Distancing during the COVID-19 Pandemic
Source: Int J Environ Res Public Health. 2021 Apr 24;18(9):4517. doi: 10.3390/ijerph18094517 (PMC8123175; doi:10.3390/ijerph18094517)

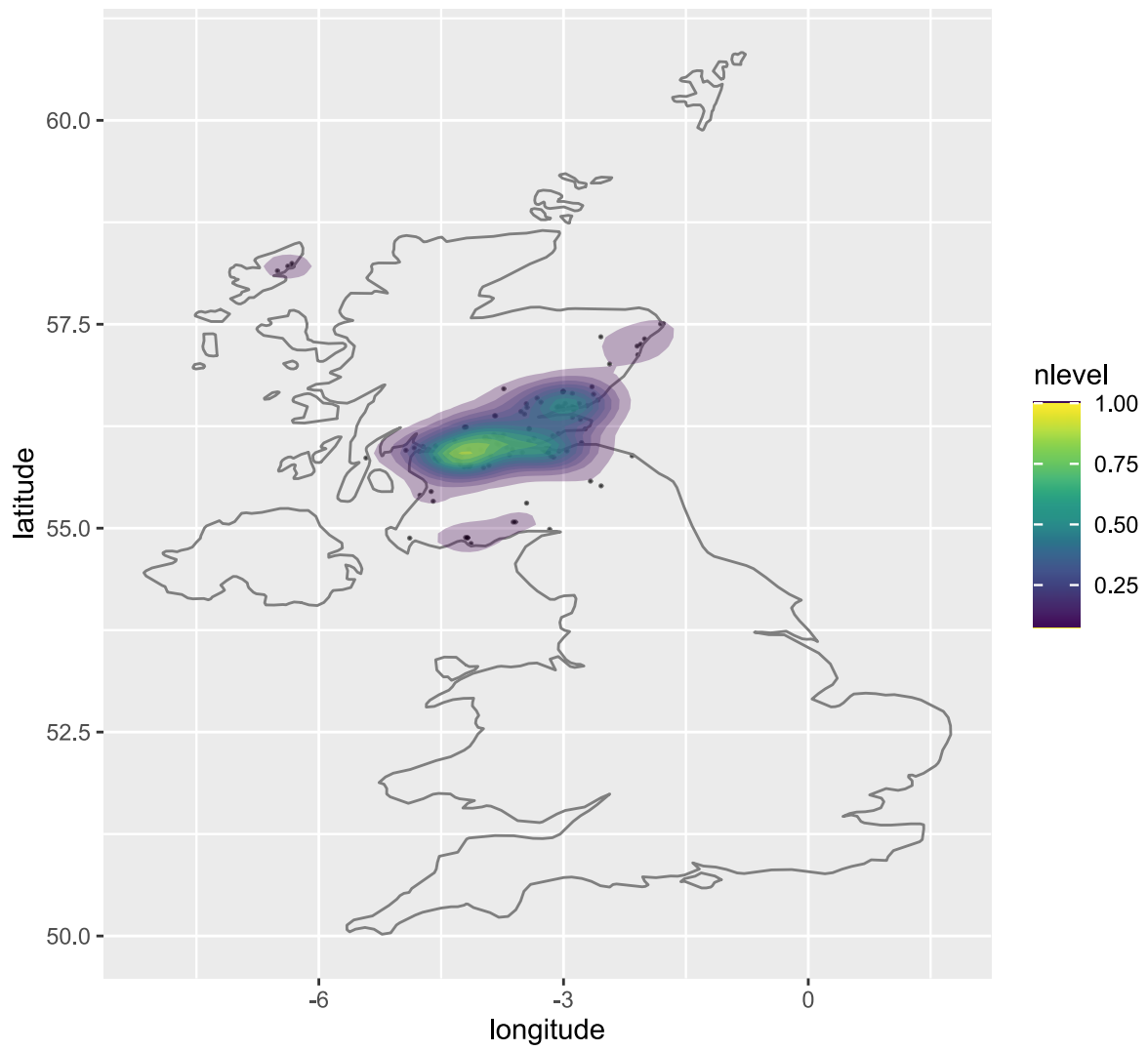

**Figure S1.** Two-dimensional density of respondents' locations across Scotland.

Supplement: Supplementary file 1 [file ijerph-18-04517-s001.zip › FigureS1.pdf]
